# Novel insights into bat influenza A viruses

**DOI:** 10.1099/jgv.0.000927

**Published:** 2017-09-14

**Authors:** Kevin Ciminski, Thiprampai Thamamongood, Gert Zimmer, Martin Schwemmle

**Affiliations:** ^1^​ Institute of Virology, Medical Center - University of Freiburg, 79104 Freiburg, Germany; ^2^​ Faculty of Medicine, University of Freiburg, 79104 Freiburg, Germany; ^3^​ Spemann Graduate School of Biology and Medicine, University of Freiburg, 79104 Freiburg, Germany; ^4^​ Faculty of Biology, University of Freiburg, 79104 Freiburg, Germany; ^5^​ Division of Virology, Institute of Virology and Immunology, CH-3147 Mittelhäusern, Switzerland

**Keywords:** influenza A viruses, bats, reassortment, zoonosis, packaging, glycoproteins

## Abstract

In 2012 and 2013, influenza virus genome sequences of two new influenza A virus (IAV) subtypes were discovered in bat specimens, but further characterization was largely impeded by the lack of infectious virus. With the identification of highly susceptible cell lines, reconstitution of infectious bat IAV by reverse genetics recently succeeded and allowed a first insight into the life cycle of these viruses. Although there is a certain degree of functional compatibility between bat and conventional influenza A virus proteins, there are striking differences, including receptor usage, polarity of infection and reassortment potential.

## Abbreviations

AIV, avian influenza virus; HA, hemagglutinin; IAV, influenza A virus; NA, neuraminidase; NCR, non-coding regions; vRNA, viral RNA; vRNP, viral ribonucleoprotein; VSV, vesicular stomatitis virus.

## Introduction

Influenza A viruses (IAVs) are the causative pathogens of annual epidemics and sporadically occurring pandemics responsible for substantial morbidity and mortality in the human population [1]. The main antigenic determinants of IAV are the surface glycoproteins haemagglutinin (HA) and neuraminidase (NA). Based on the gene homology and antigenic properties of HA and NA, IAVs have been classically subdivided into 16 different HA (H1 to H16) and nine different NA subtypes (N1 to N9) [2]. All known IAV subtypes have been found in aquatic waterfowl, which is therefore thought to represent the natural IAV reservoir [3]. Nevertheless, avian IAVs (AIVs) have recurrently crossed species barriers thereby establishing new lineages in a wide variety of hosts, including sea mammals, domestic animals such as pigs and horses, domestic poultry, and humans [3]. Here, migrating aquatic birds are of particular importance for the emergence and evolution of novel influenza viruses with zoonotic potential, since they carry all known IAV subtypes and can disseminate AIV over long distances [3, 4]. Currently, the IAV subtypes H1N1 and H3N2 are circulating within the human population, causing annual epidemics. However, there is some concern that AIV subtypes such as H5N1 and H7N9 may adapt to the human species [5, 6]. Since the human population is immunologically naïve with respect to these ‘foreign’ HA subtypes, successful establishment of a new lineage in the human population may eventually result in a pandemic associated with high mortality and morbidity. Furthermore, as IAV is a segmented negative-strand RNA virus, co-infection of pigs with avian, human and/or porcine IAV could lead to the emergence of new IAVs harbouring a novel constellation of the eight genome segments [7–10]. These so-called reassortant viruses have a high zoonotic potential, as recently exemplified by the 2009 pandemic H1N1 virus [7].

The previous understanding of the IAV host range and diversity has recently been challenged by the discovery of two novel influenza subtypes in Central and South American bat species. In 2012, a new influenza virus genomic sequence was identified in frugivorous yellow-shouldered bats (*Sturnira lilium*) in Guatemala and provisionally designated as H17N10 [11]. Only one year later, a distinct influenza genome was isolated from the flat-faced fruit bat (*Artibeus planirostris*) in Peru and initially classified as H18N11 [12]. Serological surveys in Central and South American bat populations revealed a broad prevalence of H17/H18-specific antibodies [12], suggesting a widespread circulation of these viruses in different bat species. Unfortunately, isolation of infectious virus from various bat tissues failed, greatly impeding further characterization of these new IAV subtypes. However, phylogenetic analysis of these bat-derived influenza virus genomes indicated that most genes were most closely related to those of conventional IAV [11, 12]. Notable exceptions were the putative bat-derived HA and NA genes, which showed only low homology with their counterparts from other IAVs. As outlined below in detail, biochemical data have convincingly demonstrated that the surface glycoproteins of both H17N10 and H18N11 are strikingly different from conventional IAV since they lack the canonical receptor-binding and receptor-destroying activities of conventional HA and NA, respectively [12–16]. Based on these marked differences, we suggested renaming the bat IAV surface glycoproteins as HA-like (HL) and NA-like (NL) [17].

The identification of bat IAV expanded the host reservoir of IAV and immediately raised the question of their zoonotic potential. Only recently, it became possible to reconstruct infectious bat IAV from synthetic DNA using reverse genetic approaches [18]. In this review, we recapitulate recent findings on cell tropism, entry processes and the ability of these bat IAVs to exchange genetic information with conventional IAV.

## Bat IAVs fail to reassort with conventional IAVs

The IAV genome comprises eight single-stranded viral RNA (vRNA) segments in negative polarity, each encapsidated by multiple copies of the viral nucleoprotein (NP) and terminally bound by the viral RNA polymerase, forming the viral ribonucleoprotein (vRNP) complex [19–22]. All vRNA segments share a similar structure in that they encompass a central coding region, flanked on both sides by short non-coding regions (NCR). Located at the extreme 3′ and 5′ genome ends, 12 and 13 nucleotide-long complementary sequences are highly conserved between different IAV segments and serve as promoters for the viral polymerase [23–26]. In cells co-infected with different parental IAVs, exchange of viral genome segments can give rise to IAV with a new genetic composition, a process called reassortment [7, 9, 10, 27]. The exchange of genome segments between IAVs allows rapid adaption to new host environments and is known to be responsible for the emergence of several pandemic IAV strains in the past [7, 8]. An important prerequisite for genetic reassortment is the functional compatibility of the parental viral proteins [28]. However, segmentation of the viral genome concurrently complicates vRNP packaging as it demands a highly sophisticated mechanism that coordinates the selective packaging of the eight distinct vRNPs into viral progeny particles [23]. Although the precise mechanism of IAV genome packaging into progeny virions has not been conclusively clarified yet, there is considerable evidence that each vRNA segment bears so-called packaging signals within the 3′ and 5′ NCR together with adjacent parts of the vRNA coding region [29]. It is believed that the packaging signals mediate the formation of a bundle of eight different vRNPs by forming RNA–RNA or RNA–protein interaction networks [30–33].

**Fig. 1.**
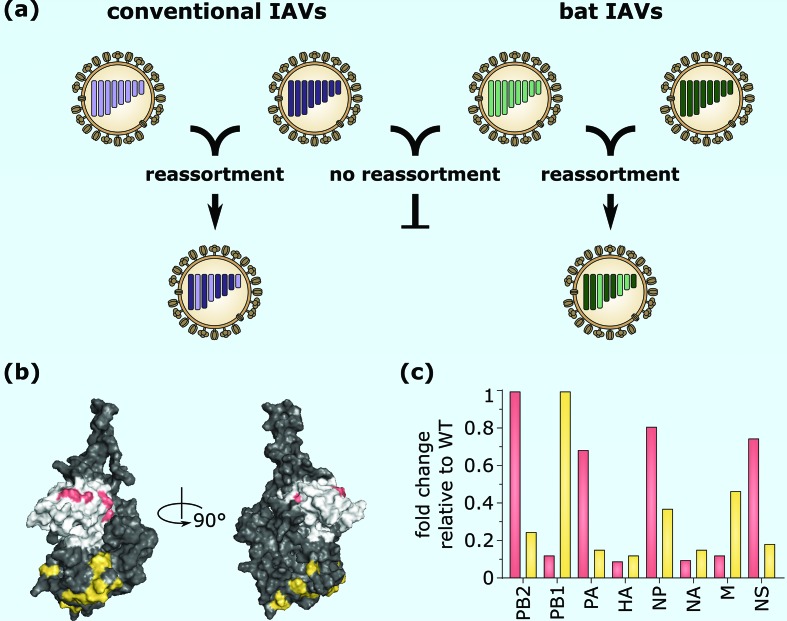
Co-infection of cells with bat and conventional influenza A viruses does not result in reassortment events. (a) The exchange of genomic segments is known to take place in cells co-infected with different conventional IAV subtypes. Likewise, based on experiments with chimeric bat influenza viruses, genomic reassortment is believed to occur in cells co-infected with the known bat IAV subtypes. However, reassortment between conventional and bat IAVs is blocked, probably due to incompatibility of the vRNA packaging signals and NP. (b and c) Highly conserved amino acids located in either the head (grey) or body (black) domain of a conventional IAV NP of the H7N7 subtype (A/SC35M) were partially substituted with the corresponding amino acids present in the HL17NL10 NP (b). Bat-specific amino acids introduced in the head or body domain are highlighted in pink or yellow, respectively. H7N7 viruses encoding these NP mutants demonstrated a genome packaging deficiency (c). The incorporation of different RNA segment subsets into viral particles was differentially affected, depending on whether the mutations were introduced into the head or body domain. The relative ratio of genome subsets identified in viral particle preparations is shown (raw data from Moreira *et al.* [40]). Genome levels of wild-type (WT) H7N7 were set to 1. Column colours correspond to the amino acid substitutions in either the head or body domain.

Bat IAVs exhibit the characteristic genome structure of conventional IAVs. They are composed of eight vRNA segments in anti-sense orientation including terminal NCRs. Likewise, the highly conserved complementary promoter sequences comprising 12 and 13 nucleotides at the extreme 3′ and 5′ ends of each bat genome segment are almost identical to those found in conventional IAVs [11, 12]. Moreover, with the exception of the envelope glycoprotein homologues HA, NA and M2, the amino acid sequences of the remaining bat virus proteins are akin to known IAV proteins reaching 50–80 % identity, depending on the protein sequence analysed. To determine the reassortment potential between bat and conventional IAV, chimeric bat viruses were engineered containing six bat gene segments from either HL17NL10 or HL18NL11 virus, along with the IAV HA and NA genes from the prototypic IAVs A/Puerto Rico/8/1934 (H1N1) (PR8), A/swine/Texas/4199-2/1998 (H3N2) or A/SC35M (H7N7) [34, 35]. These chimeric viruses were shown to efficiently replicate in various cell culture models and in mice. Of note, generation of these bat chimeric viruses by reverse genetics was only possible if the HA and NA coding regions were flanked with the authentic bat IAV NCR and parts of the coding region of the corresponding segment, already suggesting genome-packaging incompatibilities between bat influenza RNA segments and those of conventional IAV [34, 35]. Indeed, co-infection experiments confirmed that bat chimeric viruses failed to reassort with the conventional IAV subtypes H1N1, H3N2 and H7N7. However, gene reassortment was observed after co-infection with both HL17NL10- and HL18NL11-based bat chimeric viruses (Fig. 1a) [35]. In contrast to co-infection experiments, reverse genetics rescue experiments using the eight genome segments resulted in a significant higher number of reassortment events, since competition of homologous vRNA segments from different viruses for genome packaging was avoided. This might also explain the successful generation of recombinant HL17NL10-based bat chimeric reassortant viruses harbouring the HA and NA surface protein genes of PR8 plus an unmodified M segment from either PR8, A/swine/Texas/4199-2/98 (H3N2) or A/swine/Kansas/11-107824/2011 (H3N2) [36]. These reassortant viruses replicated efficiently in various cell lines and in the lungs of infected mice. Based on these findings, it is likely that the M segment packaging signals are at least partially compatible between bat and conventional IAV. However, the failure of bat chimeric viruses to reassort with conventional IAV was also partly due to an incompatibility between the internal proteins. Whereas NP of bat virus origin, and to a certain degree also the polymerase subunit PB2, supported the polymerase activity of the H1N1, H2N2, H3N2, H5N1 and H7N9 subtypes in polymerase reconstitution assays, PB1 and PA did not [34, 35, 37]. In addition, as the bat IAV non-structural protein 1 (NS1) was previously shown to share the dsRNA-binding property and IFN-suppression characteristics of conventional IAV NS1s [38], an infectious recombinant PR8 virus encoding the NS1 but not NEP gene of HL17NL10 could be generated [39]. Thus, the combination of both incompatible packaging sequences and incompatible viral proteins has likely contributed to the failure to exchange genetic information between bat and conventional IAVs.

Surprisingly, although NP of bat IAV fully supported the polymerase activity of conventional IAV of various subtypes, including H7N7, in a polymerase reconstitution assay, the generation of recombinant H7N7 viruses encoding bat IAV NP repeatedly failed, despite flanking the bat IAV NP coding region with authentic packaging sequences. However, partial substitution of H7N7 NP amino acid residues with the corresponding ones from HL17NL10 NP was tolerated and allowed the generation of viable H7N7 NP mutant viruses. Unexpectedly, these NP amino acid substitutions caused impaired viral infectivity due to an uncoordinated packaging of genomic segments into viral particles [40]. Mutational studies further revealed that as few as seven amino acid substitutions are sufficient to cause these packaging defects. Depending on whether the highly conserved H7N7 NP amino acids of the head or the body domain had been substituted with bat IAV amino acids (Fig. 1b), the incorporation of specific RNA genome segment subsets was differentially affected (Fig. 1c) [40]. This is the first indication that a viral protein can specifically recognize vRNA packaging signals, suggesting a complex interplay between NP and the vRNA packaging signals. Therefore, the lack of reassortment between bat and conventional IAV can be ascribed to the incompatibility of the vRNA packaging signals and NP. The vRNA packaging signals and the NP proteins of bat and conventional IAV might have evolved differentially in their respective hosts and probably have undergone co-evolutionary adaptation resulting in an optimal interaction of the eight distinct vRNPs during the genome packaging process. Hence, reassortment of the newly discovered HL17NL10 and HL18NL11 subtypes with conventional IAV and emergence of pandemic reassortant viruses seems rather unlikely.

## Reconstitution of authentic bat IAV by reverse genetics

**Fig. 2.**
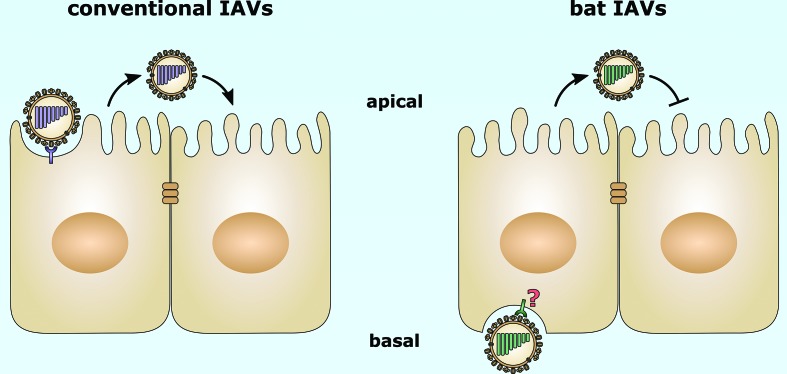
Bat and conventional IAVs exhibit different entry mechanisms in polarized epithelial cells. Conventional IAVs initiate the infection at the apical site of epithelial cells by receptor-mediated endocytosis after the binding of HA to sialic acid containing oligosaccharides exposed on the host cell surface. Following completion of the IAV replication cycle, progeny virions are then released from the apical plasma membrane where NA facilitates the cleavage of sialic acid residues. In contrast, bat IAVs enter polarized epithelial cells preferentially from the basolateral site via endocytosis upon binding of HL to a yet unknown receptor. Budding of progeny bat IAV virions occurs similarly to conventional IAV at the apical plasma membrane.

The initial failure to generate infectious bat IAVs from synthetic DNA by reverse genetics was suspected to be related to the use of non-susceptible cell lines. This problem has recently been overcome by using vesicular stomatitis virus (VSV) encoding either HL-17 or HL-18 (VSV-HL) in place of the VSV-G protein [18]. Screening of more than 30 cell lines from various species using these chimeric viruses led to the identification of Madin–Darby canine kidney type II (MDCK II) cells as being most susceptible, whereas the related MDCK I cell line turned out to be resistant to infection. In agreement with this observation, both recombinant HL17NL10 and HL18NL11 could be successfully propagated in MDCK II but not MDCK I cells. However, both MDCK cell types, which originated from the kidney of the same dog but differed in their passage history, are well known to support viral growth of conventional IAV. Subsequent studies with both chimeric VSV and recombinant HL18NL11 revealed differences in the entry mechanism compared to conventional IAV. In polarized MDCK II cells, infection by HL18NL11 was initiated most efficiently from the basolateral site (Fig. 2), a property that is shared by other viruses including VSV, hepatitis B virus, hepatitis C virus, adenovirus types 2 and 5, vaccinia virus and measles virus [41–46]. In contrast, conventional IAVs are known to enter polarized epithelial cells via the apical site of the plasma membrane [47]. The preferential basolateral infection of polarized epithelial cells by bat IAV suggests that the putative cellular receptor(s) for these viruses are preferentially expressed at the basolateral membrane but absent from the apical plasma membrane of MDCK II cells. At least two human cell lines, U-87 MG glioblastoma and SK-Mel-28 malignant melanoma cells, were found to be susceptible to infection with VSV-HL17 and VSV-HL18 as well as with authentic HL17NL10 and HL18NL11 [18]. However, whether this already indicates a zoonotic potential of bat IAVs remains to be shown. Surprisingly, recombinant bat IAVs were unable to infect cell lines from various bat species, including *Sturnira lilium* and *Artibeus planirostris,* which are believed to be naturally infected by these viruses (unpublished data). The reason for this unexpected resistance is unknown, but might be caused by the downregulation of the virus entry receptor(s) upon *in vitro* culturing of the bat cells. This was previously described for bat coronaviruses, where susceptibility or resistance of bat cell lines correlated with the presence or absence of the cell surface-expressed receptor CD26, also known as dipeptidyl peptidase 4 (DPP4) [48].

## Haemagglutinin-like molecules play crucial roles in bat IAV entry

Based on the analysis of the primary sequences, classical HA molecules are categorized into two phylogenetic groups, with group 1 comprising subtypes H1, H2, H5, H6, H8, H9, H11, H12, H13 and H16 and group 2 comprising subtypes H3, H4, H7, H10, H14 and H15 [2, 49, 50]. The number of identical amino acids in different HA subtypes varies from 40 to 70 %, and within the same subtype from 80 to 100 %. In contrast, the amino acid sequences of the bat IAV HLs are highly divergent from all known HA subtypes derived from conventional IAVs. While both HL17 and HL18 have only 45 % amino acid identity to the 16 other known HA subtypes, they share 62 % amino acid sequence identity among each other. The primary amino acid sequences and structure analysis of these proteins revealed that these glycoproteins are more closely related to group 1 than to group 2 [11, 12].

Despite the low amino acid sequence identity with conventional HA, the overall structures of HLs and conventional HAs are highly similar. The HLs also form a homotrimer containing a membrane-proximal stem region and a membrane-distal globular head, which contains the putative receptor-binding site (RBS) [12, 14, 16]. Similar to most conventional HA subtypes, HL17 and HL18 harbour a monobasic cleavage site upstream of a highly conserved stretch of hydrophobic amino acids [12, 14, 16]. At this site, the HL proteins are cleaved by serine proteases such as trypsin or human TMPRSS2, resulting in the formation of two disulfide-linked subunits, HL1 and HL2 [51]. The proteolytic cleavage of HL proteins is essential for bat IAV to be infectious. Only following proteolytic cleavage were HL proteins able to exhibit low pH-triggered membrane fusion activity. This feature is shared by bat and conventional IAVs and suggests that all of these viruses enter host cells by receptor-mediated endocytosis.

The HL proteins of bat IAVs were found to mediate autonomous propagation of HL-pseudotyped VSV without the need of support by NL proteins [18]. These chimeric viruses were able to propagate in susceptible cell lines such as canine MDCK II and bat IndFSPT cells, whereas recombinant VSV expressing exclusively the NL protein in place of VSV-G did not replicate autonomously. Interestingly, propagation of chimeric VSV was not enhanced when the corresponding NL protein was expressed along with HL. In contrast, chimeric VSV expressing conventional HA was able to propagate only if NA was expressed from the same virus genome, in agreement with the function of NA as a receptor-destroying enzyme [52]. The important role of HL proteins in the entry of bat IAV was subsequently confirmed by neutralizing HL17NL10 and HL18NL11 infection with antibodies specifically directed to HL17 and HL18, respectively [18]. On the contrary, the role of NL in the bat IAV replication cycle remains a mystery. The function of this envelope protein obviously deviates from the classical role of conventional NAs in virus release and dissemination.

## Haemagglutinin-like molecules do not recognize sialic acids as receptor(s)

The receptor-binding specificity of conventional HAs has been studied in great detail. HA proteins from avian IAVs preferentially bind to α2,3-linked sialic acids, whereas HAs of human IAVs have a preference for sialic acids in α2,6-linkage [53–55]. In principle, all the classical HA RBSs consist of two parts, designated as edge and base. The edge portion is formed by three α-helices (the 130-loop, the 190-helix and the 220-loop) while the base portion comprises only four conserved residues (Y98, W153, H183 and Y195), which mediate the binding specificity of HA [56]. Intriguingly, the crystal structure of HL17 and HL18 revealed that, in contrast to conventional HA, there is no cavity present which could accommodate sialylated glycans. In conventional IAV HA proteins, hydrogen bonds and salt bridge networks formed by the residues D136, Q190, H226 and D228 enable strong interactions between the α-helices of the edge [12, 14, 16]. According to studies on the electrostatic surface potential, the putative RBS of HL is highly acidic, making binding to negatively charged sialic acid residues unlikely. Consistently, glycan micro-array analysis revealed no detectable binding of recombinant HL17 and HL18 to more than 600 different glycan structures [12, 14].

All available data indicate that HL proteins recognize cellular receptor(s) that are dissimilar from sialic acid, the receptor determinant for conventional IAV. Interestingly, removal of sialic acids from cell surface glycoconjugates by sialidase treatment somewhat enhanced the infection of MDCK II cells by chimeric VSV-HL18, as well as by recombinant HL17NL10 or HL18NL11 [18]. The precise explanation for this phenomenon is not known, but removal of negatively charged sialic acids from the host cell surface probably facilitated binding of HL proteins to the cell surface by abrogating repulsion between sialic acid residues linked to cellular receptor glycoproteins and the negatively charged amino acids of the putative HL receptor-binding domain. In line with this proposed mechanism, a previous study showed that treatment of susceptible cells with tunicamycin, an inhibitor of N-glycosylation, reduced infection with HL-pseudotyped VSV [57], suggesting that the unknown cellular receptor is a glycosylated protein.

The extraordinary restricted cell tropism of chimeric VSV-HL and recombinant bat IAV suggested that the putative cellular receptor(s) that are recognized by HL17 and HL18 are not ubiquitously expressed. Nevertheless, they seem to be expressed by cells from different species and tissues [18, 52, 57]. Recombinant HL17NL10 and HL18NL11 showed a similar cell tropism suggesting that HL17 and HL18 bind to the same or a very similar receptor. In order to gain comprehensive insight into the entry mechanism of bat IAVs and to understand their extraordinary restricted cell tropism, it is imperative to identify the putative receptor(s) that mediate bat IAV binding to the host cell surface. The knowledge of susceptible cell lines such as MDCK II and the availability of infectious recombinant bat IAV and chimeric VSV-HL will undoubtedly facilitate the identification of receptor molecules. This in turn will advance our understanding of the potential host range and zoonotic potential of bat IAVs.

## Neuraminidase-like molecule of bat IAVs

NA of conventional IAV exhibits sialidase (neuraminidase) activity and catalyses the hydrolysis of the glycosidic linkage between a sialic acid residue and the penultimate sugar of oligosaccharides. In this way, NA destroys the receptor determinant of conventional IAV and facilitates the release of progeny virus. In addition, removal of sialic acid residues from the viral glycoproteins prevents virus aggregation. Finally, NA may help conventional IAV penetrate the sialic acid-rich mucin layers covering respiratory epithelial cells [58]. Based on phylogenetic analysis, nine subtypes of NA are distinguished and are subdivided into two groups. N1, N4, N5 and N8 belong to group 1 while N2, N3, N6, N7 and N9 are members of group 2 [59]. The NA homologues of bat IAV are only distantly related to the NAs of conventional IAV. Therefore, these NAs were primarily defined as NL10 and NL11 and were proposed to form a new group 3 [60].

Conventional NAs are type II homotetrameric membrane proteins with a mushroom-like conformation. The head domain of each NA monomer consists of six topologically β-sheets arranged in a propeller-like structure [61]. Likewise, the overall three-dimensional structures of NL10 and NL11 share striking similarity with those of classical NAs [12, 13, 15]. In addition, a calcium-binding site, which is normally required for the stabilization of the active centrum of conventional NAs, is highly conserved in both NL10 and NL11. However, the crystal structures of NL10 and NL11 revealed that the conserved amino acids normally involved in sialic acid binding are not present. Most of the amino acid residues required for NA activity are substituted and the putative active site is wider because the 150- and the 430-loops have been displaced. In agreement with these observations, sialidase activity is not associated with NL10 or NL11 [12, 13, 15] and no evidence was found for binding of NL10 or NL11 to any carbohydrate structure [12, 15]. Consequently, NL proteins might have a unique function distinct from the known sialidase activity of canonical NAs. The recent identification of susceptible cell lines and the availability of reverse genetics systems for bat IAV will certainly help in identifying the function of NL proteins in the viral life cycle.

## Conclusions

Bats represent a natural reservoir for several pathogens including Ebola virus, Hendra virus, rabies virus and SARS and MERS coronaviruses. The identification of two new influenza A-like viruses, HL17NL10 and HL18NL11, has expanded the number of potentially zoonotic IAVs. This raises important questions with respect to the origin and evolution of IAV and poses public health concerns. Recent findings demonstrated that the likelihood of reassortment between conventional IAVs and bat IAVs is limited. The inability to exchange viral RNA segments is restricted by RNA packaging signals and a matching NP. Although the risk of reassortment is low, the potential of adaptation to other mammalian hosts cannot be excluded at present. The reconstitution of synthetic HL17NL10 and HL18NL11 viruses marked a milestone in bat IAV research and allowed for the first time the characterization of the mechanisms of virus entry and cell tropism. Even though HL molecules play crucial roles in bat IAV entry, the replication cycle of bat IAVs may not follow the canonical life cycle of conventional IAVs. Moreover, the ability to infect human cell lines suggests that transmission of these viruses to the human population is still a possibility. The use of recombinant bat IAV for experimental infection of bats will provide deeper insight into their tropism and transmission model in the natural host. Importantly, identification and characterization of cellular receptors mediating virus entry will undoubtedly lead to clarification of the tissue tropism and zoonotic potential of these viruses.
